# Factors influencing intention to apply spatial approaches to on-farm experimentation: insights from the Australian winegrape sector

**DOI:** 10.1007/s13593-022-00829-w

**Published:** 2022-09-14

**Authors:** Xinxin Song, Katherine J. Evans, Robert G. V. Bramley, Saideepa Kumar

**Affiliations:** 1 Tasmanian Institute of Agriculture, University of Tasmania, Private Bag 98, Hobart, TAS 7001 Australia; 2 CSIRO, Waite Campus, Glen Osmond, SA 5064 Australia

**Keywords:** Adoption, Practice change, Precision agriculture, Spatial variation, Vineyard trials, Viticulture, Theory of Planned Behavior

## Abstract

Grape growers are often constrained by available time and labor to conduct trials that deliver informative results. Spatially distributed trial designs coupled with data collection using sensing technologies can introduce efficiencies and also account for the impact of land variability on trial results. Various spatial approaches have been proposed, yet how farmers perceive them is largely unknown. We collaborated with four wine businesses in Australia to explore how grape growers and viticultural consultants perceive a simplified spatial approach to experimentation involving one or more vineyard rows or “strips.” In each case, the simplified strip approach was applied alongside growers’ or consultants’ own methods to compare the perceived value of different methods. The Theory of Planned Behavior was used as an analytical framework to identify factors influencing participants’ intentions towards adopting the strip approach. Our findings show that growers and consultants perceived several advantages of the strip approach over their own methods. Key factors impeding uptake were resource constraints for collecting trial data and lack of skills and knowledge to use and analyze spatial data to position the trial and interpret results. These constraints highlight the need to support growers and consultants who see value in this approach by developing automated and affordable measurements for viticultural variables beyond yield, and by providing training on how to analyze and interpret spatial and response data. This study provides novel insights for private and public sectors on where to focus efforts to facilitate adoption of spatial approaches to On-Farm Experimentation by specific target audiences.

## Introduction

On-Farm Experimentation (OFE) is an important and frequently used means by which grape growers learn how alternative practices perform in their vineyards and build viticultural knowledge, thus enhancing their capacity for adaptive farm management (Hagmann and Chuma 2002; Song et al. 2022). Unlike the “specialist-enabled” OFE described by Lacoste et al. (2022), growers’ own OFE does not necessarily involve external assistance. However, while acknowledging the utility of trials to their businesses, growers are often constrained by limited time and labor to apply treatments, and collect and analyze data, thus having limited capacity to conduct trials that generate results that are useful to them (Song et al. 2022). Also, spatial variability can confound trial results or create uncertainties in translating results from a small trial area to other areas of the vineyard block (Bramley et al. 2005, 2013). These issues can limit the value of growers’ experimentation in terms of generating useful information and informing management decisions.

Spatial approaches to OFE can address these issues by accounting for the effects of land variability on trial results and improving efficiencies of data collection using sensors (Bramley et al. 2013, 2022). These approaches can be implemented using farmer-operated equipment augmented with technologies such as yield monitoring, global navigation satellite systems, and geographic information systems (GISs). Trial data can be analyzed using geostatistical methods that visualize crop responses at different locations in a production area (Bramley et al. 2013; Cook et al. 1999; Doerge and Gardner 1999). Most spatial approaches use field-scale designs that require large amounts of measurements and specialized skills for data analysis (Bramley et al. 2005, 2011; Cook et al. 1999; Milliken et al. 2010; Pringle et al. 2004). Currently, few viticultural response variables, apart from fruit yield and vine vigor, are amenable to automated measurements, although this situation is likely to change as new sensing technologies are developed. Another issue is that the required analytical methods are generally beyond the capabilities of most grape growers and their consultants, making the approaches difficult for them to implement without involving specialists.

Simple trial approaches that use one or a few strips within a field have been proposed to address some issues associated with whole-field approaches (Lawes and Bramley 2012; Whelan et al. 2012). For example, Lawes and Bramley (2012) developed a simple approach in which a trial strip is located across pre-determined management zones and moving window *t*-tests are applied to reveal spatial variation in treatment differences along the strip. Information of how crop responses vary along the strip and in different management zones can, in theory, inform a farmer’s decision as to whether to apply crop inputs uniformly or differentially, or create new management zones in the field. The analysis can be completed by farmers and consultants with the aid of a spreadsheet.

Little attention, however, has been given to farmers’ perception of the various spatial approaches to OFE or their intention to adopt them. One exception is a case study in North America by Griffin et al. (2008) who found that experiments using spatial approaches increased farmers’ confidence in management decisions and triggered farmers’ interest in learning spatial data analysis for their future experiments. Researchers who conducted whole-of-block experiments in Australian vineyards (Lanyon and Bramley 2006; Panten and Bramley 2012; Panten et al. 2010) reported that the results improved vineyard managers’ knowledge about the impact of land variability on vine performance. There is a need to identify and understand what aspects of spatial approaches are valued, or not, by farmers and to understand the factors that influence their intentions towards adoption. Such knowledge can then inform interventions that facilitate uptake of these approaches by farmers and their consultants.

**Fig. 1:**
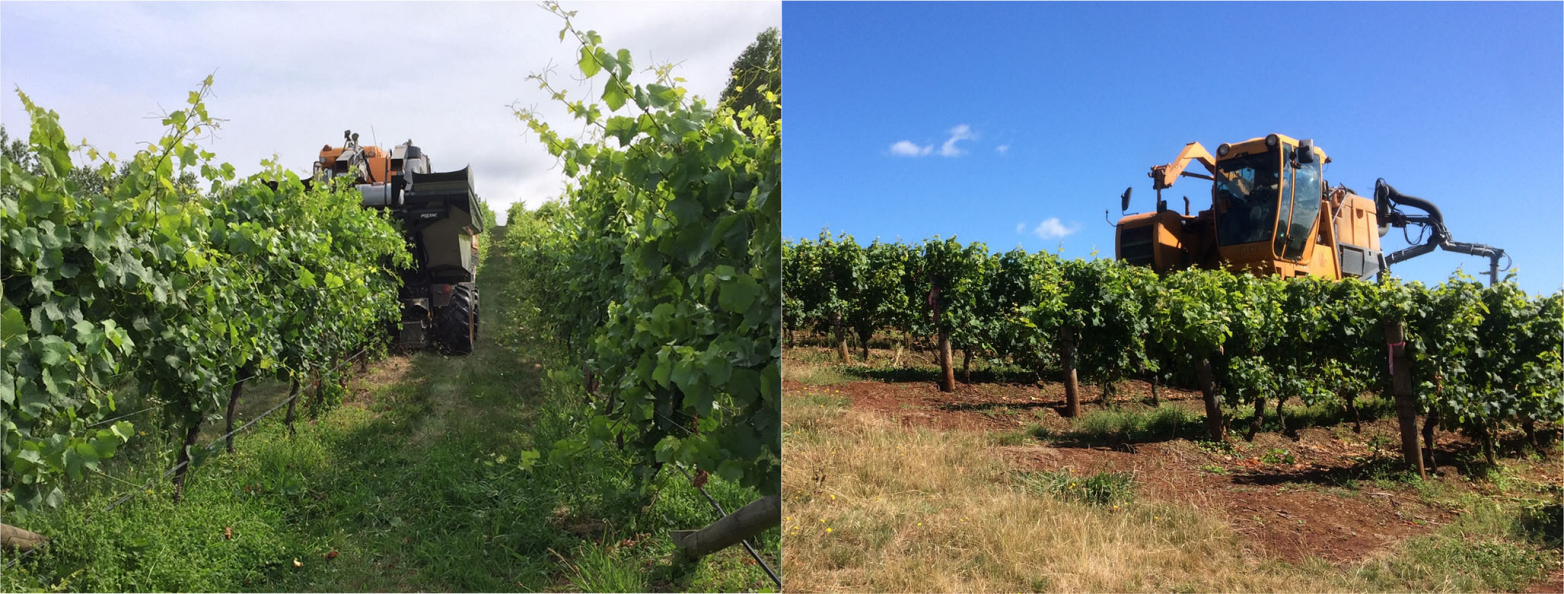
Collaborative trials where vineyard staff applied treatments using their own equipment. Photographs by Xinxin Song.

The aim of this study was to identify factors influencing the intentions of grape growers and consultants in Australia to adopt a strip trial approach modified from that described by Lawes and Bramley (2012). The factors were identified from their perceptions about the approach. Perception in this study refers to the opinion or belief that growers and consultants form about the strip approach in terms of its application in their specific commercial farming context. The strip approach was applied to vineyard trials (Fig. 1) conducted by the research team, four growers and two consultants. The intent was to help wine businesses answer questions that are unique to their business and operational context by introducing efficiencies and robustness to farmers’ experimentation. Ajzen's (1985) Theory of Planned Behavior (TPB) was used as an analytical framework to examine the perceptions of the growers and consultants about the approach. We discuss the significance of the findings in relation to potential improvements to spatial approaches to OFE and the types of support needed by growers and consultants intending to adopt these approaches for their own experiments.

## Materials and methods

The authors collaborated with four wine businesses in three states of Australia on vineyard trials (Table 1) using a participatory approach (Carberry 2001). In each case, the grower set the objectives for the trial (Table 1). The authors worked with the growers and consultants as equal partners from trial design through to interpretation of trial results. The growers and consultants implemented the co-designed trial in the vineyards; they also collected trial data for the strip approach with the support from the first author except in case 4 in which the first author undertook data collection. The first author analyzed the data of the strip trial while the grower or consultant analyzed the data collected using their own methods (see below). The authors, grower, and consultant interpreted trial results together. As such, the growers and consultants implemented the trial within their operational capacity and evaluated the strip approach based on their own situation and information needs. All the trials were conducted to investigate how growers and consultants would perceive the use of a spatial trial approach for their own OFE. The growers and consultants participated because they were interested in trying a new trial method.

**Table 1:** Key details of collaborative trials with four wine businesses in Australia. ^a^Digital elevation model indicating elevation (m); ^b^Normalized difference vegetation index of crop vigor; ^c^Plant cell density, a vegetation index of crop vigor (Dobrowski et al. 2003). More information about the trials is provided in Song (2022).

	Case 1: mechanical shaking trial	Case 2: netting trial	Case 3: compost trial	Case 4: biological spray trial
Trial objective	Evaluate the effect of mechanical shaking before bunch closure on the severity of botrytis bunch rot (caused by *Botrytis cinerea*) at harvest	Assess the effect of white, green and black netting against no netting on color of grape bunches. Bunch color has been associated with wine flavors in this region (Deloire et al. 2017)	Evaluate the effect of compost application on winter pruning weights of vines	Compare the effect of three different spray programs for the control of botrytis bunch rot
Trial site	A 2.2-ha block of Sauvignon Blanc grapes in the Tamar Valley, Tasmania	A 4.5-ha block of Chardonnay grapes at Orange, New South Wales	A 5.2-ha block of Cabernet Sauvignon grapes at Coonawarra, South Australia	A 1.2-ha and a 2.1-ha block of Riesling grapes in the Tamar Valley and Pipers Brook, Tasmania
Treatments	(1) No shaking; (2) mechanical shaking before bunch closure using a commercial harvester	(1) No net; (2) white net; (3) Green net; and (4) black net applied to vine rows before bunch closure to until harvest	(1) No compost; (2) compost applied under vines before budburst	(1) Standard program: fenhexamid and cyprodinil; (2) standard spray with *Bacillus amyloliquefaciens* applied at pea-size; (3) standard spray with *Bacillus amyloliquefaciens* applied 4 weeks before harvest
Response variable	Mean botrytis severity per vine (%) near harvest	Mean bunch color indicated by hue near harvest	Pruning weight (kg/vine) in winter	Mean botrytis severity per vine (%) near harvest
Candidate covariate and spatial data	DEM^a^	• DEM^a^ • NDVI^b^ in 2020	• DEM^a^ • PCD^c^ in 2020, 2021	DEM^a^
Participant and role in wine business	Grower G1: vineyard manager working as an employee	Grower G2: the business owner; consultant C1: industry development officer from a state government agency	Grower G3: vineyard manager working as an employee	Grower G4: vineyard manager working as an employee; consultant C2: agronomist from a company supplying agronomic products
Trial period	2019–2020	2019–2020	2019–2021	2020–2021

The participants in cases 1–3 were selected from a list of growers and consultants who participated in a survey conducted by Song et al. (2022), while those in case 4 were identified from the networks of the second author. The growers had similar production goals aimed at improving the uniformity in grape yield and berry composition. Thus, it was assumed that the strip approach would be relevant to them. The variation in their trial objectives and methods allowed us to test the strip approach in different contexts. The participants were all male, aged between 30 and 64 years, and had a diploma degree or above in agriculture or viticulture, which Song et al. (2022) showed was representative of growers and consultants in the Australian wine sector. All the participants in this study had more than 10 years’ experience in viticulture or agriculture while G3 also had professional experience involving the use of spatial data and GIS. All wine businesses were family-owned, with the businesses of cases 1 and 3 having a corporate management structure. The total vineyard area managed by the growers ranged from 80 to 330 ha. The growers performed varied roles in their businesses (Table 1) and were responsible for the management of the vineyard including trial-related work. Both of the consultants were external to the businesses (Table 1). Specifically, C1 was employed by a state government and provided unpaid advice to G2; C2 provided paid advice to G4 as part of a “package” with purchased agronomic products.

The trial design of each case used a modified strip approach, hereafter referred to as the “simple strip” approach, whereby trial strips are positioned in a block such that the range of variation in a potentially useful covariate to a response variable of interest in the strips is close to that encountered over the whole block. Hence, the strips can, in theory, generate information about likely variation in the response variable across the block, thus better informing management decisions. Useful covariates were determined by their correlation to a response variable of interest where such data were available; or trial locations were selected according to all available spatial data, with useful covariates being identified when trial data were collected. The data were analyzed using a moving window comparison (Lawes and Bramley 2012) without involving statistical tests due to the complexity of accounting for spatial auto-correlation and the fact that statistical significance is not considered important by grape growers for their decision-making (Song et al. 2022).

**Fig. 2:**
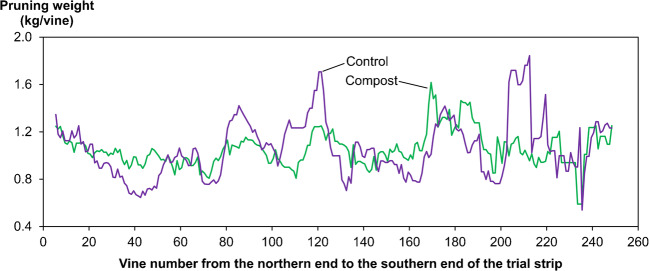
Spatial variation in pruning weight (kg/vine) for compost and control treatments along the trial strip in the 2019–2020 growing season for the compost trial of case 3.

The collaborative trials were conducted to answer the questions that grower participants had for their vineyards (Table 1). The shaking trial of case 1 was positioned according to elevation, a useful covariate identified for the severity of Botrytis Bunch Rot (BBR) at the trial site; two strips encompassed 85% of the range of variation in elevation in the whole block. The trial of case 4 was also positioned according to elevation, on the assumption that it was a useful covariate to botrytis severity at this site. However, the trial locations of cases 2 and 3 were determined by the growers and consultant to meet other trial objectives or to reduce risks of production loss and input costs; they did not consider candidate covariates. Nonetheless, the strips encompassed a certain range of variation in candidate covariates. In each case, data collection and analysis were done using two approaches—the method normally used by the grower or consultant, and the simple strip approach introduced by the authors. For the latter, measurements of crop responses were conducted at every fourth vine in trial strips for case 1, every third vine for cases 2 and 4, and every vine for case 3 according to logistical feasibility of each case. The growers’ or consultants’ own methods produced the mean effect per treatment; the authors’ method identified spatially varying treatment effects along trial strips (e.g., Fig. 2).

The first author conducted face-to-face semi-structured interviews (interview guide provided in Appendix) with each participant before and after each trial. The interview before the trial covered business context, participants’ experience in viticulture, experimentation, and precision agriculture (PA). After the trial was completed, the research team and participating growers and consultants jointly discussed and compared trial results produced using the strip approach and their own methods, that is, a line graph (e.g., Fig. 2) versus an average value per treatment. Reflections were also made on the implementation of the trial methods. Following the discussion or at a later date, interviews were conducted with the participants individually to obtain their perceptions of the strip approach, along with their intentions to adopt the strip approach. Where an interview could not be undertaken in person due to COVID-19 restrictions, an online video meeting was used as a substitute. Each interview lasted between 1 and 3 h. All interviews were audio-recorded and fully transcribed for analysis. Notes of field observations and casual conversations during the trials and email exchanges between the researchers and participants were also used as qualitative data.

Thematic analysis of all the qualitative data was carried out using the software, NVivo (QSR 2020). Ajzen’s (1985) TPB, described below, was used as an analytical framework for data analysis. Codes associated with quotes of interviewees reported in this paper reflect the role of an interviewee in a wine business (G, grower; C, consultant).

## Analytical framework for adoption intentions

In this study, we used TPB as an analytical framework; that is, we applied it to qualitatively analyze participants’ perceptions about the strip trial approach and factors influencing their intentions to adopt or adapt the approach. In-depth understanding of such perceptions and intentions was deemed important to help improve the strip approach and support its adoption. There are various theories for examining factors influencing the adoption of technology or the process of adoption, such as the diffusion of innovations (Rogers 2003); the capability, opportunity, motivation, and behavior model (Michie et al. 2011); and adoption pathway analysis (Montes de Oca Munguia et al. 2021). However, we considered them not suitable for this study because, for example, they are focused on technologies of interest or different stages of adoption. Conversely, we found that TPB was appropriate for explaining why a person intends to, or not, adopt a particular technology.

**Fig. 3:**
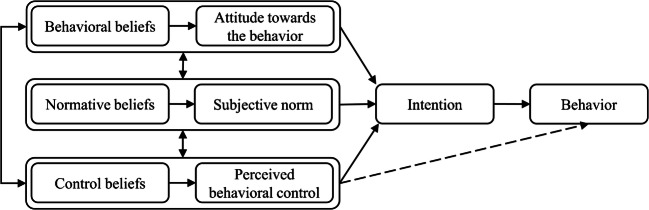
The Theory of Planned Behavior model (Ajzen 1985).

The participants’ perceptions of the strip approach were grouped according to the three constructs of TPB (Fig. 3): attitudes, subjective norms, and perceived behavioral control. In this study, attitudes refer to positive or negative evaluation of the strip approach; subjective norms include the opinions of peer growers, the operational context and opinions of other staff or owners in the business regarding the use of the strip approach; and perceived control is the perceived efficacy or ability to implement the strip approach. These constructs were determined by corresponding antecedents: behavioral beliefs, normative beliefs, and control beliefs that the participants developed about the strip approach (Fig. 3). According to TPB, a person will have a strong intention to perform a certain behavior if they evaluate the behavior positively, believe that they are supported to perform it, and perceive that they have the capacity for performing it. While attitude, subjective norms, and perceived control jointly affect intention, their relative importance tends to vary with different behaviors, individuals, and situations (Ajzen 1985). Perceived control can directly influence behavior (dotted line in Fig. 3) because it can also be a measure of actual control, such as resource availability, provided that the person has sufficient information to identify the control factors (Ajzen 1985).

The efficacy of TPB for studying human intention and behavior has been supported by five decades of research in a broad range of disciplines, including health (McEachan et al. 2011), marketing and consumer behaviors (Arvola et al. 2008; Vermeir and Verbeke 2008), ecology (Kaiser et al. 1999), and agriculture (Fielding et al. 2008; Hall et al. 2019; Hansson et al. 2012). Importantly, the qualitative use of TPB can provide in-depth insights on factors influencing technology adoption and on processes of decision-making, as demonstrated by some studies in various research domains, such as consumer behaviors, education, and agriculture (Cheng 2019; Hall et al. 2019; Kelleher et al. 2016; King and Dennis 2006; Sutherland 2010, 2011).

TPB, of course, has its limitations and has received some criticisms. A common criticism is that TPB is confined to behavior with rational reasoning and it fails to account for the influence of emotions, identities, or other factors on human behavior (Manstead and Parker 1995; Miller 2017; Rapaport and Orbell 2000; Wolff et al. 2011). However, this is a misinterpretation of the model. As Ajzen and Fishbein (2000) pointed out, TPB does not assume that people behave in a rational manner. In fact, people’s beliefs about a given behavior can be developed from inaccurate information and biased by emotions such as disgust or fear (Ajzen 2011). The beliefs, regardless of how they are formed, will naturally lead to attitudes, subjective norms, and perceived control which then influence intentions and actions. Another issue frequently raised about TPB is that its three constructs are insufficient for predicting or explaining people’s intentions and behaviors (Conner and Armitage 1998; Manstead and Parker 1995). However, rather than being rigid, the model has always been open to additional variables or predictors (Ajzen 2011). For example, Wolff et al. (2011) added “uncertainty avoidance” to the model to better predict the intention towards genetic testing. Despite the criticisms, the referred literature largely supports the explanatory power of TPB for behavioral intentions.

## Findings

Findings are organized according to the three constructs of TPB (Table 2; Fig. 3), recognizing that they can influence each other and so are not mutually exclusive.

**Table 2:** A brief summary of the findings organized according to the three constructs of the Theory of Planned Behavior (Ajzen 1985). ^a^Reference refers to the number of coded texts in NVivo.

TPB constructs			Themes		References^a^
Attitudes	The strip approach design	Positive attitudes	Easy, practical		7
			More efficient		4
			Representative		4
		Negative attitudes	Confusing		1
			Counter intuitive		1
			Irrelevant		1
	Data collection	Positive attitudes	Less biased		7
			Quick to do		4
			Feasible to do		2
			Capture variation		1
		Negative attitudes	Time, labor intensive		17
			Stressful		2
	Moving window comparison	Positive attitudes	Arouse curiosity		4
			Easy to do		3
		Negative attitudes	Time demanding		2
			Statistical significance		2
	Trial results	Positive attitudes	Increase confidence		15
			Insights on variation impact		13
			Food for thought		11
			Inform better management		10
			Simple to interpret		7
			More rigorous		3
		Negative attitudes	Limited value		8
			Limited relevance		9
			Confusing interpretation		8
Subjective norm			Current vineyard management strategy		22
			Current use of imagery		10
			Communication with peers		3
			Opinions regarding Precision Agriculture		2
Perceived control			Resource availability	Time	20
				Staff	18
				Budget	5
			Skill and knowledge	Spatial data analysis	18
				Moving window comparison	8
				Positioning trial strips	3
				Interpreting results	1
			Obtaining spatial data		10
			Using results for targeted management		10
			Vine surveying		2

### Attitudes towards the simple strip approach

#### Perceived robustness of trial results

Participants perceived that the strip approach generated more rigorous and informative results compared to their own methods, thereby increasing their confidence in decision-making. Note that in some cases, the trials did not produce meaningful results for management decisions; for example, all treatments for BBR in cases 1 and 4 produced similar results because seasonal weather conditions were conducive to extremely high disease severities. Even so, participants believed that the strip approach would likely produce useful results under less extreme weather conditions.

The systematic sampling of the strip trial was viewed as advantageous over random sampling in that it could better capture variation in land and enable less biased results, which then could contribute to management decisions. G3 noted “it has increased the confidence in aspects or thoughts that I would have had in managing the vineyard and certain, like features of the property… therefore I’ll be more confident in future seasons in what I’m doing.” The detailed results of varying crop responses could contribute to important decisions: “if I’ve got solid data like that, we can make those bigger value financial decisions… and [if] there’s a significant financial outlay, we’ll probably want a bit more rigor than what we’ve got” (G3). G2 believed that “if you persist with using technology to identify the variations, that this is where we’re going in the future. And that the old random plotting across the block using mathematical formula will have its day very soon.”

Variation in botrytis severity along trial rows changed G4’s belief in the value of mean treatment effects: “you could draw conclusions based on those averages [of botrytis severity]. Yeah, but I’m not going to because I can see that there’s that variation... I can’t draw a lot of conclusions from the numbers that we’ve captured this year on an average basis then.” The mean botrytis severity for each treatment of the spray trial could have led to a flawed decision of adopting treatment 2 that had the lowest mean severity. However, the strip approach showed that it was less effective than other treatments in many locations of the trial strips.

On seeing spatial variation in treatment effects, C2 indicated the risk of discrediting himself when advising growers based on his own results: “from a commercial point of view, I might, if I’m spreading biologicals [fungicides] to a client, this makes it quite difficult for me to do that without having, um a crisis of confidence in my, am I selling it to sell a product or am I selling it to sell that answer to a problem? If you were to do that you know you could end, I could end up exposing my credibility you know.” Therefore, C2 expressed an intention to change practice: “I would change the way I do assessment now… because it makes sense to me that that is a more statistically useful technique,” and “I already think that I need to talk to my colleague about incorporating this moving box assessment in the way she assesses her trials as a way to make it more weighty.”

While the rigor of the strip approach was appreciated, the absence of statistical tests for the moving comparisons might be a concern for consultants who would need higher levels of confidence than growers to disseminate trial results or advice growers.

#### Perceived value for understanding variation

All the growers perceived the strip approach positively, stating that it confirmed or improved their understanding of the influence of spatial variability on crop responses. They observed variation in crop responses along the strips and associated it with variation in the covariates involved and their knowledge of the sites. Growers were aware that the land underlying the vineyard is non-uniform and that spatial variation can confound the interpretation of trial results. G2 commented: “you’re giving me a chance to look at some of the major variants that are in a vineyard and trying to relate that to the effect of netting… We always question the effect of elevation and vigor on these types of results, by putting that map [DEM] in, we’re getting to eliminate it.” They indicated that the improved understanding may improve vineyard management: “one would like to think that, that would lead to improvements in management more broadly or just understanding a block better, which, invariably, you would have to say leads to improvements, be they efficiency, quality or whatever they may be” (G3).

#### Perceived value for targeted management

In the view of growers, variation in crop responses along strips combined with information about variation in covariates could inform targeted management in a block. Most participants, however, saw high-resolution variation in treatment effects as not necessarily useful to them. One reason was limited capability for efficient targeted management. For example, G1 pointed out the difficulty of turning information to action: “look, that’s [targeting individual panels or vines] too complicated for us, too micro on a larger scale thing... Quite often like we’ll get information and we’ll get results that spark us thinking about different approaches but, trying to implement something is then the hard bit... It’s a matter about what we can action without causing too much labor intensity.” This challenge was echoed by C2: “it’s always the same discussion I have with clients is that I say, ‘well we can generate a really nice map [showing variation in a variable of interest], but how can we use that information to make change?’”

Another reason for limited usefulness perceived of varying crop responses was uncertain benefits and costs of targeted management. As G1 noted: “for us to go any lower than a block, there’s gotta be benefit to it. So, if we’re gonna start looking at individual vines or individual rows, how is that gonna be better for us in the long run?” While acknowledging that current management could be improved, G4 noted: “we don’t have the information to tell us that it’s... there is benefit in changing our program any more than we’ve got it now or being more specific than what we’ve got... But we know it’s not good enough, but we don’t know where to go yet to improve it. So, until we get those answers, there’s probably not a great deal of sense in changing it to... and working with averages is probably okay.”

Growers were willing to apply targeted management when simple application was enabled and when they were convinced of the benefits. For example, G1 and G3 had applied varying compost within blocks using automation tools along with maps prescribing where to vary inputs. While G1 perceived limited value of trial results for targeted management, he acknowledged the value of the information as it may be actionable in future: “it might take a technical advancement in equipment for me to be able to treat that differently. So if I could get a leaf plucker that, a variable leaf plucker that automatically changes rate to a density map or something like that then, then I’ve got a way to do it.”

Different from other participants, G3 placed significant value on high-resolution data of pruning weight per vine because the information provided him with flexibility to manage at different levels of resolution according to different needs: “I believe I need to be managing data to the individual vine level, because I can go down to that level, I can also scale up from that level.” Moreover, G3 was forward-looking and proactive in terms of collecting data and expected to derive value from it to give the business a competitive advantage: “who knows what new sensors will come online and what new measures we’ll have. Like could it be that we’ll be using new vine indices to measure performance in future or that existing indices could be collected more easily? It’ll be important to be in a position to be able to capture, store and analyze data to be able to act quickly if necessary.”

#### Perceptions about the design

Growers appreciated the efficiency of the strip design in that it could save time and reduce costs by using a small trial area, such as one or a few rows, and then extrapolate results to a large area, such as a whole block. G2 explained: “if we can do the strip trials to allow us to have variation in the block from a scientific point of view, but do it in one row, that’s spot-on mate… So there are two major things, it will save time, save cost of damage. And the other thing would be that it allows you to expand the experiment more which allows for every dollar spent. You can get a bigger result from a smaller area.”

While G1 valued the efficiency of the strip approach, he also felt that his method of sampling vines across the block may provide a more complete overview of variability in the block. This grower then added that for the strip trial to be representative, the number of strips should be proportional to the size of a block: “maybe if it was one row an acre block or hectare block or something like that would work. But if it’s a five-hectare block, you’d need to do five rows.” This may suggest a need for sufficient information about factors that influence a response variable in a given trial area so that growers have confidence in the positioning of the trial and results. Also, growers may need to compare the strip approach with their own methods for several seasons to better learn about its usefulness before deciding to adopt the approach or not.

Interestingly, while appreciating the approach, C2 pointed out that the idea of incorporating variation in trials was counter-intuitive because it was against their previous knowledge and practice of dealing with variation in OFE: “intentionally selecting the most variability, which is probably counter-intuitive to what you’d perceive to be the right way to do it because you’ve been sort of geared towards thinking, if you reduce the variables then, it will give you a better understanding of the performance [of treatments].”

Incorporating spatial information in trial strips was not necessarily relevant to C1 as he tended to remove variation from a trial for the purpose of generalizing results beyond a vineyard hosting the trial. Instead of using imagery, C1 preferred to rely on growers’ knowledge to find a trial location directly relevant to his aim of a trial. Nonetheless, seeing the results of moving window comparisons sparked his interest in applying the analysis method to another trial, although he was uncertain about what would be achieved by doing it: “well, I’m not too sure at the moment… but I’ll be able to see if certain areas of the row are, you know visually standing out for... you know is it elevation, is it different soils, those type of things. I would be able to, um look at it in a different way than just doing an ANOVA [analysis of variance] based-off replicates within a row.”

#### Perceptions about spatial data

Perceptions of the value of different types of spatial data were explored. G2 and G3 highly valued spatial data describing, for example, elevation, vine vigor, and soil properties, and had obtained them for most of their blocks to guide management decisions. In comparison, G1 and G4 only saw information about variability in vine vigor as useful. They saw little value in DEM or yield maps as they felt that they could obtain that knowledge in other ways, such as walking through the site, or were uncertain about the value of the data to them. Consultants used spatial data infrequently in their work, although C2 believed that the data would be increasingly important to him as more farming businesses apply them for operational decisions.

The growers, except G3, perceived little need to re-purchase new vigor imagery because the pattern of variation was largely temporally stable: “so, I’ve got other areas to spend money than just re-doing imagery for the sake of having a map that’s only two years old, and that’s telling the same story from 10 years ago” (G4). G3 was aware the issue, attributing it to limited value derived from the imagery: “you know if more value isn’t made of the aerial imagery, the broad trends don’t change and that’s why a lot of growers could be saying, ‘well I know where the high, low and medium vigor areas are… I feel comfortable in my understanding, well I don’t need to get the imagery anymore…’.” G3 then indicated potential value in extracting information from spatial data over time: “…which is again a reason why I wanna be able to extract more data and raw data from the imagery so I can actually start making, getting that temporal understanding.”

### Subjective norms about trial approaches

#### Influence of business context

Business context and practices were found to affect growers’ trial behaviors to some extent. The business G1 worked for had developed their own trial protocols and the collection and documentation of numerical data were encouraged. G1 explained: “we actually do document it a bit, a lot more than just me saying ‘yes it worked, no it didn’t work’ sort of thing. So, you’ve gotta have some data behind it generally… They wanna see that the time that we’re investing in these things, we are actually having outcomes, whether they be positive or negative.” Nonetheless, G1 had autonomy to change approaches so long as trial information was collected, documented, and reported in the business management system. The other businesses had no specific requirements for trial approaches and growers usually designed trials based on their own knowledge and resources available.

All wine businesses supported the use of spatial data in managing vineyards, especially the businesses of G2 and G3. In fact, G2 believed that the use of spatial data would become a standard operating procedure for farming businesses. The company that employed C2 and some of his colleagues viewed spatial data skills and knowledge as important for keeping abreast of recent advances and informing advice given to clients, as C2 explained: “I had a colleague who told me if you don’t get on board with this stuff, you’ll be superseded in 10 years’ time because that’s where it’s headed. So you kind of, as a business, we have to value it because, yeah, there’s intersect with what we do.”

#### Influence of peer pressure

Growers reported little evidence of peer pressure influencing their own trial methods. When chatting about trials with other growers, such as neighbors, their interest was mostly focused on questions and results of a trial. They rarely discussed approaches to trials. Conversely, consultants were more likely to discuss trial methods with each other. Also, consultants were expected by farmers to experiment in a practical way and provide relevant and reliable results.

### Perceived behavioral control about the strip approach

#### Perceptions about applying the design

Participants viewed using one or a few strips to trial as easy and practical: “I like it because it’s... I can simply explain that to someone to go and apply different treatments. If you get your randomized block design type of trials, and you’ve got four panels and then another four different panels, another four different, you’re back to where you were. That’s really hard to get people to do stuff amongst” (G4). This might be partly why participants often used adjacent rows for a trial. C1, however, perceived positioning trials according to variation in a covariate as confusing. When looking at vigor variation in the block indicated by NDVI and discussing where to conduct the netting trial, C1 commented: “I look at that now and it starts to confuse me of where I should go.”

#### Resource constraints for data collection

Participants identified limited resources for data collection, including time, labor, and finance, as a major obstacle to adopting the strip approach. Resource constraints are an everyday challenge for vineyard managers who often prioritize operations according to immediate needs. G3, who found it stressful to manually prune every vine and measure pruning weights for the compost trial, commented: “vineyard operations are already running quite ‘lean’ in terms of staff and systems.” The difficulty was echoed by G4: “so, you’re pulling labor from some part of your group that’s obviously there for a reason to come and do something else, but then something else is gonna fall aside unless you find, so it’s good to find the dollars to replace somebody or the dollars to pay for someone to do this work [data collection] and it’s a never-ending battle, right? And meanwhile we’ve got sheds out at our vineyard that are leaking rainwater every time it rains. So what’s the priority? Doing this or fixing the sheds, fixing all the iron on top of the sheds, so that our roofs don’t leak and our machineries not getting all wet every time it rains? So, there’s always demand, lots of demands, and you’ve gotta prioritize where the funds go at any point in time.” Growers also noted that staff may not have the capacity for trial work: “as capable as staff may be, they may not necessarily be technically minded or have the attention to detail potentially required to do the data collection” (G3). Training staff for trial work or collecting data by themselves would be a drain on growers’ time that could be deployed elsewhere.

G1, however, did not experience resource constraints when collecting data using the strip approach: “we flew through it, it was great.” This is because, while more samples (assessing BBR at every fourth vine) were required than G1’s method, the duration of sampling was similar and the strip approach meant that G1 did not need to walk across the entire block to collect data.

Given these constraints, G2 and G4 often relied on visual data or external assistance, such as consultants, to measure results. Even G4 valued numerical data, he often had to base decisions “on gut feel, discussing with colleagues, listening to suppliers and what they put forward.” G2, the business owner, was aware of the risk of lack of documentation: “so the risk to the business is that, it’s a pretty substantial value that I don’t remember or, worse than that, I get run over and it’s all gone.”

Automated data collection was seen as critical to make trial work more practical: “I would love if there was more data collection, but I just can’t see how that would happen without additional tools, so to automate the process, to make it a lot more streamlined” (G3). At the time of the study, affordable automated measures of viticultural variables, except for vine vigor, were not available to the vineyards. As an alternative, participants suggested reducing the amount of data to be collected without losing rigor, such as dividing a block into several zones and collecting data accordingly. However, G3 said that the resolution of such data may be less valuable to them.

Collecting position data of individual vines using a global positioning system (GPS) unit was also an issue for using the strip approach. The accuracy and reliability of GPS units that growers have access to and can afford may be problematic. This can make crop surveying difficult and time-consuming, while creating problems for analyzing data of treatment effects correlated with other spatial data. G3 noted the difficulty of surveying: “you’ve got to firstly purchase the GPS, have a GPS of the correct accuracy… Then will the GPS work on the day, to the accuracy you require. We experience that as well you know. It’s painful, it’s time-consuming, it doesn’t always work.” Moreover, using GPS to locate certain target vines was seen as difficult: “a GPS position is not necessarily practical for a grower... If you give them a block, row number, meters down the row, panels down the row or vine number down the row, that’s something you can find easily” (G3).

#### Access to affordable and suitable spatial data

Another obstacle was access to sound and affordable spatial data, especially for vineyards in regions that currently have limited access to aerial data capture or a high frequency of suboptimal atmospheric conditions. For example, a high incidence of cloudy days in Tasmania limited the use of satellite imaging during the trial of case 4 and there was no access to affordable aerial imaging. Also, while there are freely available elevation data of 1-m resolution for many wine regions in Australia, participants simply did not know how to access and use it. Additionally, trial results may be misinterpreted if spatial data contain errors introduced during data capture or there is misalignment with vineyard block boundaries.

#### Capability for data analysis

Even if suitable spatial data were available, all participants, except G3, did not feel they had skills and knowledge needed to position trial strips using covariates. G4 suggested that the absence of skillsets for spatial data was common in many wine businesses. This issue also limited growers’ use of vigor imagery to visual inspection instead of analyzing it to derive more value. G2 noted the need to understand different types of spatial data and to process the data: “we need a basic understanding of the different images that’s collected. So whether it’s NDVI, etc., etc., we need to understand which is the one that is the one we wanna use.” Additionally, G3 pointed out that there was limited industry support for growers to identify and solve problems they may encounter in applying precision agriculture.

Growers G2 and G4 said that they did not have time nor see value for them to learn: “I don’t have enough hours in the day to learn a new skill that I might use once every two or three years maybe. It’s just the value is not there” (G4). G4 felt that relying on his and his staff’s knowledge about the site may be sufficient: “you’d be surprised how much, how much specific knowledge people have in the vineyards of vineyards to be able to select somewhere to do your trial and not, perhaps, put it in the wrong spot.” However, for trials conducted to answer important questions, they might seek external assistance from consultants or research institutions.

The moving window comparison was not a significant concern, except for the time needed to perform the analyses. For example, while admitting that the analysis was convenient to do in a spreadsheet, C2 expected a tool to automate it to reduce the time needed while making it less intimidating because “data analysis... is always intimidating, I think, to people.”

#### Interpreting results

Generally, participants perceived the format of line graphs (e.g., Fig. 2) showing variation in crop responses along strips as easy to interpret. The exception was G4 who found it complicated: “that’s confusing because there is so much information there and, what you draw from it… It’s really difficult to get a simple take-home message out of it.” G4’s observation might reflect the complexity of the trial results rather than the presentation given that treatment responses (BBR severity) were highly variable within and among treated rows.

All participants favored the format of a map or image, stating that it was simple to understand and to explain to others, such as the wine maker, co-workers, or business owners. G1 explained: “it helps others understand if you’re presenting that data or trying get it across to other people. They don’t know what my block looks like, if you show them the elevation with the map overlaid, they get an understanding perhaps.”

## Discussion

This study identified factors influencing the intentions of participating growers and consultants in terms of adopting the modified strip approach for their own experimentation. While the small number of participants precludes wider generalization of the findings, many wine businesses participating in a previous study in Australia (Song et al. 2022) indicated a desire for trial approaches that free up time and labor in a highly mechanized farming sector. For those farmers and consultants who are interested in adopting spatial approaches to OFE, insights from this study point to potential private sector services and PA technologies that they might need. For agricultural innovation systems more broadly, this study highlights the value of public-private sector collaborations in revealing key factors that, if addressed, would enable wine businesses to become more self-reliant in achieving business improvements through OFE.

The growers and consultants perceived several advantages of the strip approach, including rigorous and informative results presented in a way that aids interpretation, and efficiency and ease of implementation. Growers also suggested that this approach can improve their understanding of the impact of spatial variability on crop responses, consistent with the findings of Bramley et al. (2005) and Panten and Bramley (2012). While the results produced using the strip approach may not necessarily lead to a change in management, they could contribute to grower learning by informing how land variation influences crop performance and prompting them to ask further questions. Similar findings were observed by McCown (2012) for Australian farmers’ use of decision support systems. These positive perceptions likely contributed to the intentions of participants, who had autonomy to choose their preferred trial methods, to apply the strip approach to their own trials where appropriate.

Learning and awareness can be precursors to adoption of technology. Several studies have suggested that adoption of a practice is not a one-off event or a linear process, but often occurs in a stepwise and dynamic manner (Abadi Ghadim and Pannell 1999; Montes de Oca Munguia et al. 2021; Pannell et al. 2006). Through a series of testing and learning activities, farmers reduce their uncertainties about a practice and improve their skills for applying it to their own farming systems (Pannell et al. 2006). This form of adoption was evident in our cases. For example, a consultant was interested in applying the moving window comparison to one of his trials to explore what results could be generated. A grower intended to try this strip approach but preferred to position a strip and collect data based on management zones instead of sampling every few vines. Indeed, rather than following it rigidly, growers and consultants may adapt the strip approach or use some elements to fit their own circumstances or information needs (Pannell et al. 2006).

The main barriers to adoption of the strip approach include limited resources for collecting trial data; access to sound and affordable spatial data, skills, and knowledge for analyzing spatial data; and the capacity for efficiently applying trial results for targeted management. The availability of PA technologies is critical for farming businesses to implement the strip approach and targeted management according to variation in crop response of trial results practically and efficiently. However, recent interviews with grape growers in Australia (Song et al. 2022) found that their use of PA was largely limited to vigor mapping, with soil sensing and yield monitoring used much less commonly. Also, anecdotal evidence suggests that variable rate technology (VRT) adoption in the Australian wine sector is not yet common, especially for small vineyards. In other agricultural sectors in the USA, UK, and Australia, the adoption rates of yield mapping, soil mapping, and VRT were also mostly below 50% (Llewellyn and Ouzman 2014; Lowenberg-DeBoer and Erickson 2019). Limited uses of particular elements of PA may be due less to costs than to unclear value propositions for farmers (Bramley and Ouzman 2019), uncertainties about their benefits (Barnes et al. 2019; Jochinke et al. 2007), or a lack of knowledge and capabilities in sourcing the data.

Participants in this study had limited skills, knowledge, and time to process spatial data in GIS software and derive value from them. While two growers were interested in learning the skills, others had no intention to learn; instead, they might consider using an external service if necessary. This suggests a role for agricultural consultants in assisting growers with experiments using spatial approaches. Studies in Australia, New Zealand, and Germany (Ayre et al. 2019; Bramley and Ouzman 2019; Eastwood et al. 2019; Kutter et al. 2011; Llewellyn and Ouzman 2014) have reported that advisory services play an important role in supporting farmers to apply PA. Consultants could also act as intermediaries between research organizations and farmers to articulate needs, maintain linkages, and thus facilitate adoption of spatial approaches (Klerkx et al. 2012).

There are, however, several issues associated with consultants adopting spatial approaches to OFE. Capacity for GIS skills is not widespread among consultants in the Australian wine sector (unpublished data in Song et al. 2022). Given that consultants are likely to learn when there is demand from their clients (Nettle et al. 2018), growers’ demand for spatial trials can encourage consultants to learn the skills themselves or undertake training. Another issue could be beliefs consultants might hold that involving spatial variation in a trial is counter-intuitive and conflicts with the methods they have learnt. The beliefs people form about an object can influence their attitudes towards it (Ajzen 1985). As such, people who believe that trial methods should attempt to remove the effects of spatial variation on trial results may be reluctant to change their current methods. This is consistent with the “inertia” described by Cook and Bramley (2001); that is, the difficulty for consultants to change from the methods that they regard as reliable towards new methods such as spatial approaches about which they are uncertain. Further, the lack of statistical tests may lead to greater hesitation among consultants towards the strip approach given that they often need a high degree of confidence in the robustness of trial results to support advice they give to clients. However, the limited value of statistical significance in determining treatment efficacy or practical significance has been pointed out by many researchers (Amrhein et al. 2019; Wasserstein et al. 2019; Whelan et al. 2012) and farmers rarely base their decisions on statistical significance (Bramley et al. 2005, 2013, 2022; Song et al. 2022).

Farmers’ uptake and implementation of spatial approaches may also be assisted by researchers, as in the four examples in this study. Indeed, in farmer-centric OFE, researchers or other specialists co-design trials with farmers, and can help them collect and analyze data, and interpret results (Lacoste et al. 2022; MacMillan and Benton 2014), thus producing outputs that are both rigorous and relevant to farmers’ needs. As observed in this study and suggested by others (Carberry et al. 2002; Mackenzie et al. 2012), the participatory approach can enable social learning among all participants. For example, the growers and consultants learned about the strip approach while the authors gained in-depth understanding of conducting experiments in commercial farming contexts. Lessons learnt from the participatory OFE are described in detail in Song (2022). As such, there is potential for using co-innovation approaches (Botha et al. 2017) via OFE to improve the design of spatial approaches, to develop new criteria for assessing trial results, and to enhance the capacity of farmers and consultants for using spatial approaches.

Unlike most studies using TPB to predict intentions or behaviors (Sok et al. 2021), this study used TPB as an analytical framework to deconstruct and analyze participants’ perceptions about the strip trial approach. The main value of such use of TPB is that it helped the authors explore and identify the influence of attitudes, subjective norms, and perceived control on participants’ intentions to adopt the strip approach. As such, TPB enabled in-depth insights on factors influencing their behavioral intentions, thus informing where interventions can be applied to support behavioral change in farmer experimentation. Similar findings were also noted by other studies aimed at understanding and explaining farmers’ behaviors of interest (Hall et al. 2019; Home et al. 2014; Sutherland and Holstead 2014). However, not using TPB to design this research might be a reason for fewer data collected on subjective norms than on attitudes or perceived control. While this could reflect relative importance of factors in participants’ perceptions, it may also be related to the choice of interview questions. Further, this study focused exclusively on the strip approach and its use in a vineyard setting, seeking to gain deep insights about using a spatial approach for growers’ own OFE. This aim influenced selection of participants who, although having not been exposed to spatial approaches to OFE prior to our collaboration, were interested in trying such an approach. They might not represent the majority of farmers and consultants. Systems research involving a greater diversity of participants in different agricultural sectors (Klerkx and Nettle 2013; Klerkx et al. 2012) is likely to reveal broader implications about applying spatial approaches for OFE by farmers, consultants, and specialists which may then improve uptake of the approaches.

## Conclusion and implications

Application of the Theory of Planned Behavior in this study enabled the identification of attitudes, subjective norms, and control factors influencing growers’ and consultants’ intentions to adopt or adapt spatial approaches to their own OFE. Importantly, the use of this theory enabled deep understandings of why these factors are, or are not, influential. We found that the participants valued trial results using a simple strip approach incorporating spatial data and the efficiency of the approach. Even so, its intended uptake was constrained by limited resources to collect trial data, and skills and knowledge for analyzing spatial data, which are the same factors influencing the adoption of whole-field approaches. Two other factors limiting the uptake were access to quality and affordable spatial data and growers’ capacity to apply trial results for targeted management within a vineyard block. These findings contribute insights on how the public and private sectors could support farmers and consultants to adopt spatial approaches to OFE for their own trials. To do so, an important first step would be providing information about reliable sources of spatial data, along with easy access to such data. Automated measurements of viticultural response variables beyond yield and vigor data are also needed. Growers and consultants will need to learn to select among different types of spatial data for their experimentation, to use analytical tools in GIS software, and to interpret and apply trial results for improved crop management. An easy-to-follow protocol for the strip approach or other approaches can streamline the implementation by farmers and consultants. Moreover, a more useful measure of treatment differences, such as agronomic significance, as opposed to statistical significance, will be likely needed for farmers to better assess trial results. A change in consultants’ attitude towards statistical tests may also be necessary.

Given the small number of cases involved in this study, there is a need to conduct more research to test whether the findings are applicable to other farming businesses and to investigate more general situations in different farming sectors. Broader knowledge of current capabilities and capacities for spatial approaches to OFE in the farming sector will support development of training and learning programs for current and future farmers and consultants. More examples of collaborative OFE involving farmer groups, consultants, and researchers would provide further insights on adoption-related issues and digital literacies which can then inform future interventions and research efforts in the public and private sectors. Assumptions would need to be tested about the capacity and willingness of farming businesses to pay for OFE-related services and/or technologies designed to generate commercial insights and value. Co-innovation through participatory OFE can also be used to improve spatial approaches while supporting farmers and consultants to use the approaches. As spatial approaches become embedded in various farming sectors, there will be a concurrent need to update higher education curricula so that the next generation of farmers and consultants develop a solid theoretical foundation upon which to base their eventual on-ground practices.

## Data Availability

Dataset sharing not applicable to this article as we do not have the consent to share the dataset generated during the current study.

## Appendix. Interview Guide

**Interview before participatory experimentation**

Business context

1. How important is conducting trials for the business? Why?
2. Who are involved in trial activities in general?
3. What are their responsibilities in the business?
4. What are their responsibilities in terms of conducting trials?

Trial methods

1. How many years of experience do you have in conducting vineyard trials?
2. What methods do you use for trials? Why?
3. How was the method developed?

Precision Agriculture

1. How much experience do you have in using Precision Agriculture technology?
2. What types of spatial data are used in the business? Why?
3. What do you think of the value of Precision Agriculture? Why?
4. How do other people in the business perceive the technology?

**Interview after participatory experimentation**

Perceptions of the simple strip approach to experimentation

1. How do you perceive the advantage of the design of the simple strip approach compared to the design that you used?
  - Can you explain the reasons for the perceptions?
  - What about its disadvantages? Why?

(The same questions were asked in terms of data collection, data analysis and trial results of the strip approach)

2. Would you like to apply the strip approach to your trials in future? Why or why not?
3. What were the challenges you experienced in using the strip approach?
4. What support would you need to apply the strip approach to your trials in future, if you are willing to?
